# Mechanisms of Nanoparticle-Induced Oxidative Stress and Toxicity

**DOI:** 10.1155/2013/942916

**Published:** 2013-08-20

**Authors:** Amruta Manke, Liying Wang, Yon Rojanasakul

**Affiliations:** ^1^Department of Pharmaceutical Sciences and Mary Babb Randolph Cancer Center, West Virginia University, Morgantown, WV 26506, USA; ^2^Physiology and Pathology Research Branch, National Institute for Occupational Safety and Health, Morgantown, WV 26505, USA

## Abstract

The rapidly emerging field of nanotechnology has offered innovative discoveries in the medical, industrial, and consumer sectors. The unique physicochemical and electrical properties of engineered nanoparticles (NP) make them highly desirable in a variety of applications. However, these novel properties of NP are fraught with concerns for environmental and occupational exposure. Changes in structural and physicochemical properties of NP can lead to changes in biological activities including ROS generation, one of the most frequently reported NP-associated toxicities. Oxidative stress induced by engineered NP is due to acellular factors such as particle surface, size, composition, and presence of metals, while cellular responses such as mitochondrial respiration, NP-cell interaction, and immune cell activation are responsible for ROS-mediated damage. NP-induced oxidative stress responses are torch bearers for further pathophysiological effects including genotoxicity, inflammation, and fibrosis as demonstrated by activation of associated cell signaling pathways. Since oxidative stress is a key determinant of NP-induced injury, it is necessary to characterize the ROS response resulting from NP. Through physicochemical characterization and understanding of the multiple signaling cascades activated by NP-induced ROS, a systemic toxicity screen with oxidative stress as a predictive model for NP-induced injury can be developed.

## 1. Introduction

The growing field of nanotechnology has transformed many sectors of the industrial field with their breakthrough applications in the areas of biotechnology, electronics, medicinal drug delivery, cosmetics, material science, aerospace engineering, and biosensors. Manufactured nanomaterials (NM) have gained commercial interest in a variety of consumer products. Their novel physicochemical, thermal, and electrical properties facilitate their application in clothing, medicine, and cosmetics thereby increasing the probability for human and environmental contact with these NM [1–3]. Of all the NM, carbon nanotubes (CNT) and metal-based nanoparticles (NP) have generated considerable commercial interest owing to their remarkable intrinsic properties such as high tensile strength and conductivity, which in turn meet the needs of the specific application for which these NP are designed [4, 5]. Their widespread use raises concerns of their inadvertent exposure in humans and the consequent deleterious health effects [6]. As compared to the growing commercial interest of NM, modest research effort has been invested in evaluating the potential adverse effects of these engineered NM. The sheer multiplicity of the physicochemical parameters of NM such as size, shape, structure, and elemental constituents makes the investigation of their toxic effects complex and challenging [7]. Some of the paradigms for NP-mediated toxicity include oxidative stress, inflammation, genetic damage, and the inhibition of cell division and cell death [8–11]. Most work to date has suggested that ROS generation (which can be either protective or harmful during biological interactions) and consequent oxidative stress are frequently observed with NP toxicity [3, 9]. The physicochemical characterization of NP including particle size, surface charge, and chemical composition is a key indicator for the resulting ROS response and NP-induced injury since many of these NP intrinsic properties can catalyze the ROS production [6]. NP-mediated ROS responses have been reported to orchestrate a series of pathological events such as genotoxicity, inflammation, fibrosis, and carcinogenesis. For instance, CNT-induced oxidative stress triggers cell signaling pathways resulting in increased expression of proinflammatory and fibrotic cytokines [12]. Some NP have been shown to activate inflammatory cells such as macrophages and neutrophils which can result in the increased production of ROS [13–15]. Other NP such as titanium dioxide (TiO_2_), zinc oxide (ZnO), cerium oxide (CeO_2_), and silver NP have been shown to deposit on the cellular surface or inside the subcellular organelles and induce oxidative stress signaling cascades that eventually result in oxidative stress to the cell [16]. The mechanism for ROS generation is different for each NP and to date the exact underlying cellular mechanism for ROS generation is incompletely understood and remains to be elucidated. Most of the metal-based NP elicit free radical-mediated toxicity via Fenton-type reactions [4, 17], whereas mitochondrial damage plays a major role in CNT-mediated ROS generation [18]. However, it is inaccurate to assume that ROS generation is a prerequisite to NP-induced toxicity since a few studies have reported the direct toxicity of NP without causing ROS [19]. Nevertheless, ROS generation is a major event during NP-induced injury that needs to be thoroughly characterized in order to predict NP-induced toxicity. This review will focus on oxidative stress as a mechanism for understanding NP-induced toxicity. For this paper, we have considered metal-based NP and CNT in the light of oxidative stress. The relationship between different NP characteristics and resulting oxidative stress is discussed.

### 1.1. Generation of ROS

ROS, key signaling molecules during cell signaling and homeostasis, are reactive species of molecular oxygen. ROS constitute a pool of oxidative species including superoxide anion (O_2_^•−^), hydroxyl radical (OH^•^), hydrogen peroxide (H_2_O_2_), singlet oxygen (^1^O_2_), and hypochlorous acid (HOCl). ROS are generated intrinsically or extrinsically within the cell. Molecular oxygen generates O_2_^•−^, the primary ROS via one-electron reduction catalyzed by nicotinamide adenine dinucleotide phosphate (NADPH) oxidase. Further reduction of oxygen may either lead to H_2_O_2_ or OH^•^ via dismutation and metal-catalyzed Fenton reaction, respectively [20, 21]. Some of the endogenous sources of ROS include mitochondrial respiration, inflammatory response, microsomes, and peroxisomes, while engineered NM, environmental pollutants act as exogenous ROS inducers. Physiologically, ROS are produced in trace amounts in response to various stimuli. Free radicals occur as essential byproducts of mitochondrial respiration and transition metal ion-catalyzed Fenton-type reactions [20]. Inflammatory phagocytes such as neutrophils and macrophages induce oxidative outburst as a defense mechanism towards environmental pollutants, tumor cells, and microbes. A variety of NP including metal oxide particles induce ROS as one of the principal mechanisms of cytotoxicity [22]. NP have been reported to influence intracellular calcium concentrations, activate transcription factors, and modulate cytokine production via generation of free radicals [12, 23].

### 1.2. Oxidative Stress

Abundance of ROS can have potentially damaging biological responses resulting in oxidative stress phenomenon. It results from an imbalance between the production of ROS and a biological system's ability to readily detoxify the reactive intermediates or repair the resulting damage. To overcome the excess ROS response, cells can activate enzymatic and nonenzymatic antioxidant systems [24]. The hierarchical model of oxidative stress was proposed to illustrate a mechanism for NP-mediated oxidative stress [4, 9]. According to this model, cells and tissues respond to increasing levels of oxidative stress via antioxidant enzyme systems upon NP exposure. During conditions of mild oxidative stress, transcriptional activation of phase II antioxidant enzymes occurs via nuclear factor (erythroid-derived 2)-like 2 (Nrf2) induction. At an intermediate level, redox-sensitive mitogen-activated protein kinase (MAPK) and nuclear factor kappa-light-chain enhancer of activated Bcells (NF-*κ*B) cascades mount a proinflammatory response. However, extremely toxic levels of oxidative stress result in mitochondrial membrane damage and electron chain dysfunction leading to cell death. Some of the key factors favoring the prooxidant effects of engineered NM include either the depletion of antioxidants or the increased production of ROS. Perturbation of the normal redox state contributes to peroxide and free radical production that has adverse effects on cell components including proteins, lipids, and DNA [23]. Given its chemical reactivity, oxidative stress can amount to DNA damage, lipid peroxidation, and activation of signaling networks associated with loss of cell growth, fibrosis, and carcinogenesis [16, 25, 26]. Besides cellular damage, ROS can result from interactions of NP with several biological targets as an effect of cell respiration, metabolism, ischemia/reperfusion, inflammation, and metabolism of various NM [22]. Most significantly, the oxidative stresses resulting from occupational NM exposures as well as experimental challenge with various NP lead to airway inflammation and interstitial fibrosis [27–30].

### 1.3. Nanoparticle-Induced Oxidative Stress

Nanomaterials of varying chemical composition such as fullerenes, CNT, and metal oxides have been shown to induce oxidative stress [20, 31]. The key factors involved in NP-induced ROS include (i) prooxidant functional groups on the reactive surface of NP; (ii) active redox cycling on the surface of NP due to transition metal-based NP; and (iii) particle-cell interactions [22, 25]. From a mechanistic point of view, we discuss the sources of ROS based on the physicochemical parameters and particle-cell interactions.

Several studies demonstrate the significance of reactive particle surface in ROS generation [20, 32]. Free radicals are generated from the surface of NP when both the oxidants and free radicals bound to the particle surface. Surface bound radicals such as SiO^•^ and SiO_2_^•^ present on quartz particles are responsible for the formation of ROS such as OH^•^ and O_2_^•−^ [17, 25]. Ambient matter such as ozone and nitrogen dioxide (NO_2_) adsorbed on the particle surface is capable of inducing oxidative damage [16]. Reduced particle size results in structural defects and altered electronic properties on the particle surface creating reactive groups on the NP surface [27, 33]. Within these reactive sites, the electron donor or acceptor active sites interact with molecular O_2_ to form O_2_^•−^ which in turn can generate additional ROS via Fenton-type reactions [3]. For instance, NP such as Si and Zn with identical particle size and shape lead to diverse cytotoxicity responses due to their surface properties. ZnO being more chemically active than SiO_2_, led to increased O_2_^•−^ formation resulting in oxidative stress. Free radicals are either directly bound to the NP surface or may be generated as free entities in an aqueous suspension [17]. Dissolution of NP and subsequent release of metal ions can enhance the ROS response [25]. For instance, aqueous suspensions of quartz particles generate H_2_O_2_, OH^•^, and ^1^O_2_ [17, 20, 32].

 Apart from surface-dependent properties, metals and chemical compounds on the NP surface accelerate the ROS response [34]. Transition metals including iron (Fe), copper (Cu), chromium (Cr), vanadium (V), and silica (Si) are involved in ROS generation via mechanisms such as Haber-Weiss and Fenton-type reactions [25]. Fenton reactions usually involve a transition metal ion that reacts with H_2_O_2_ to yield OH^•^ and an oxidized metal ion. For example, the reduction of H_2_O_2_ with ferrous iron (Fe^2+^) results in the formation of OH^•^ that is extremely reactive and toxic to biological molecules [21]. Cu and Fe metal NP have been reported to induce oxidative stress (O_2_^•−^ and OH^•^) via Fenton-type reaction [26], while the Haber-Weiss-type reaction involves a reaction between oxidized metal ion and H_2_O_2_ to induce OH^•^ [21, 35]. NP including chromium, cobalt, and vanadium can catalyze both Fenton and Haber-Weiss-type reactions [26]. Glutathione reductase, an antioxidant enzyme, reduces metal NP into intermediates that potentiate the ROS response. In addition, some metal NP (Ar, Be, Co, and Ni) promote the activation of intercellular radical-inducing system such as the MAPK and NF-*κ*B pathways [36]. 

In addition to the prooxidant effect of NP, ROS are also induced endogenously where the mitochondrion is a major cell target for NP-induced oxidative stress. Once NP gain access into the mitochondria, they stimulate ROS via impaired electron transport chain, structural damage, activation of NADPH-like enzyme system, and depolarization of the mitochondrial membrane [37, 38]. For instance, cationic polystyrene nanospheres induce O_2_^•−^ mediated apoptosis in murine macrophages based on their ability to target mitochondria [38]. 

Cellular internalization of NP has been shown to activate immune cells including macrophages and neutrophils, contributing to ROS/RNS [22, 25]. This process usually involves the activation of NADPH oxidase enzymes. *In vivo* particle exposures such as silica activate the rich pool of inflammatory phagocytes within the lung causing them to induce oxidative outburst [39]. NP with smaller particle size are reported to induce higher ROS owing to their unique characteristics such as high surface to volume ratio and high surface charge. Particle size determines the number of reactive groups/sites on the NP surface [34, 37, 40]. The pulmonary responses induced by inhaled NP are considered to be greater than those produced by micron-sized particles because of the increased surface area to particle mass ratio [28]. Larger surface area ensures that the majority of the molecules are exposed to the surface than the interior of the NM [3]. Accordingly, nano-sized SiO_2_ and TiO_2_ and MWCNT induce greater ROS as compared to their larger counterparts [41]. Additionally, a study with cobalt/chromium NP exposure demonstrated particle size dependent ROS-mediated genotoxicity [42].

## 2. Oxidant Generation via Particle-Cell Interactions

Besides being self-oxidative in nature, NP react with cells and induce their prooxidant effects via intracellular ROS generation involving mitochondrial respiration and activation of NADPH-like enzyme systems [43]. NP can activate the cellular redox system specifically in the lungs where immune cells including alveolar macrophages (AM) and neutrophils act as direct ROS inducers. Professional phagocytic cells including neutrophils and AM of the immune system induce substantial ROS upon internalization of NP via the NADPH oxidase enzyme system [44]. The phagocytic oxidative outburst is attributable to some of the NP physicochemical properties. In case of silica and quartz particles, inflammation-induced ROS was associated with the surface-based radical-generating properties of the particles [45]. Additionally, NP from the residual oily fly ash and diesel exhaust activate the pool of inflammatory phagocytes resulting in massive ROS release [46]. Furthermore, adsorption of chemicals such as organic matter onto the NP surface may drive the inflammation-induced oxidative stress [24].

### 2.1. Lung Injury Caused by Nanoparticle-Induced Reactive Nitrogen Species

Besides oxidative damage, NP exposure within the lung is reported to induce reactive nitrogen species (RNS). Particle deposition in the lung causes recruitment of inflammatory cells that generate ROS, clastogenic factors, and cytokines either harming or stimulating resident lung cells [31]. Inflammatory phagocytes are an important source of RNS/ROS generation within the lung. Owing to their inducible nitric oxide synthase (iNOS) activity, phagocytes can produce a large amount of genotoxic RNS, including nitric oxide (NO^•^) and the highly reactive peroxynitrite (ONOO^−^). ONOO^−^ formed by the reaction of NO^•^ and O_2_^•−^ causes DNA fragmentation, lipid oxidation, and protein dysfunction consequently contributing to particle-induced lung injury [47]. *In vivo* exposure to SiO_2_ and quartz NP elicited an RNS response characterized by increased iNOS and NO^•^ within the lung as a result of phagocyte influx [48, 49].

### 2.2. Mechanisms of ROS Production and Apoptosis within Metal Nanoparticles

Apoptosis has been implicated as a major mechanism of cell death caused by NP-induced oxidative stress [50–52]. Among the different apoptotic pathways, the intrinsic mitochondrial apoptotic pathway plays a major role in metal oxide NP-induced cell death since mitochondria are one of the major target organelles for NP-induced oxidative stress [38]. High levels of ROS in the mitochondria can result in damage to membrane phospholipids inducing mitochondrial membrane depolarization [53]. Small proportion of electrons escapes the mitochondrial chain and interacts with molecular oxygen to form O_2_^•−^ which later gives rise to H_2_O_2_ or partially reduces to the damaging OH^•^. NP can catalyze the O_2_^•−^ generation either by blocking the electron transport chain or accelerating electron transfer to molecular oxygen [54, 55]. Various metal oxide NP including Zn, Cu, Ti, and Si elicit ROS-mediated cell death via mitochondrial dysfunction [56–59].

## 3. Introduction to Transition Metals

Transition metal oxide particles have been used to revolutionize several fields including catalysis, sensors, optoelectronic materials, drug delivery, automobile, and material science engineering. Apart from industrial scale applications, metal NP are increasingly used in a variety of consumer products such as cosmetics, sunscreens, textiles, and food products. Among the transition metal oxides, titanium dioxide, cupric oxide, and zinc oxide have gained attention owing to their commercial usage [60]. Metal oxide particles can undergo surface modification for better stability and binding to other substrates. Such widespread applications are attributable to their electrochemical and physical properties reflecting their small sizes and reactive surfaces. For example, a relatively inert metal or metal oxide may become a highly effective catalyst when manufactured as NP. Their fixed particle mass, high aspect ratio, and particle surface bioreactivity tailor them to meet the needs of specific application. However, a high surface-to-volume ratio makes NP reactive and exposes them to environmental stressors, particularly free radical generation [61, 62]. Besides, the nanoscale dimensions enhance their cellular uptake and interaction with biological tissues. Metals can generate free radicals via the Fenton-type reactions that react with cellular macromolecules and induce oxidative stress [63]. The toxicity of metallic NP including Zn, Ti, Si, Fe, and Ce has been characterized by increased ROS generation and oxidative stress and apoptosis [61, 64–66]. The oxidative stress mediated outcomes of various metal NP are summarized in Table 1.

## 4. Prooxidant Effects of Metal Oxide Nanoparticles

To overcome the overwhelming ROS production, cells trigger either a defensive or an injurious response eliciting a chain of adverse biological responses. Free radicals are potentially damaging to cellular macromolecules including lipids, proteins, and nucleic acids. DNA is one of the major targets for oxidative stress and represents the first step involved in mutagenesis, carcinogenesis, and aging. ROS/RNS cause oxidative DNA damage in the form of DNA strand breaks, DNA protein cross-links, and alkali-labile sites [67, 68], and given their characteristic nature free radicals appear as one of the likely carcinogens [25, 69]. Testing the genotoxic potential is essential for carcinogenic risk assessment of NP. Genotoxic effects may be produced either by direct interaction of particles with genetic material or by secondary damage from particle-induced ROS. Transition metal NP induce chromosomal aberrations, DNA strand breaks, oxidative DNA damage, and mutations [70]. OH^•^, one of the highly potent radicals, is known to react with all components of DNA causing DNA single strand breakage via formation of 8-hydroxyl-2′-deoxyguanosine (8-OHdG) DNA adduct [71, 72]. 8-OHdG is a biomarker of OH^•^-mediated DNA lesions. NP exposure significantly elevated 8-OHdG levels both *in vivo* [73] and *in vitro* [74], demonstrating their mutagenic behavior. A recent study comparing metal oxide NP including Cu, Fe, Ti, and Ag reported ROS-mediated genotoxicity characterized by micronuclei and DNA damage *in vivo* [75].

Along with chromosomal damage, free radicals also interact with lipids and proteins, abundantly present in biomembranes, to yield lipid peroxidation products associated with mutagenesis. Polyunsaturated fatty acids are subject to oxidation giving rise to lipid hydroperoxides as the initial step in ROS generation [25, 76]. Prooxidant metals such as Cu and Fe react with these lipid hydroperoxides to induce DNA damaging end-products malondialdehyde (MDA) and 4-hydroxynonenal that act as inflammatory mediators and risk factors for carcinogenesis. Exposures to metal oxide NP of Ti, Cu, Si, and Fe were reported to induce tissue damage, abnormal cellular stress response via lipid peroxidation [77–79].

Alterations within the antioxidant defense system pose as a risk factor for carcinogenesis [68]. Glutathione, (GSH) a potent free-radical scavenger, is responsible for maintaining the cellular redox state and protecting cells from oxidative damage [80, 81]. NP-triggered free radicals reduce GSH into its oxidized form glutathione disulfide (GSSG), thereby contributing to oxidative stress, apoptosis, and sensitization to oxidizing stimuli [82, 83]. Apart from GSH, NP-induced ROS modulate the antioxidant activities of ROS-metabolizing enzymes including NADPH-dependent flavoenzyme, catalase, glutathione peroxidase, and superoxide dismutase [84]. 

It is well established that uncontrolled generation of ROS triggers a cascade of proinflammatory cytokines and mediators via activation of redox sensitive MAPK and NF-*κ*B signaling pathways that control transcription of inflammatory genes such as IL-1*β*, IL-8, and TNF-*α* [21]. Oxidative stress plays a key role in NP-induced airway hypersensitivity and respiratory inflammation [85]. A study involving coexposure of metal oxide NP with a bacterial endotoxin demonstrated exaggerated lung inflammation and pulmonary edema [86]. Additionally, studies with different metal oxide NP have demonstrated ROS-mediated inflammatory response. For instance, SiO_2_ and TiO_2_ NP induce an elevated inflammatory response through the underlying mechanism of ROS generation [64, 85, 87]. Pulmonary inflammation may induce changes in membrane permeability, facilitating NP distribution beyond the lung and indirectly affecting cardiovascular performance [88]. 

Metal ion-induced free radicals can activate oncogenes such as Ras [25]. Excess amounts of NP have been associated with skin, bladder, liver, lung, and respiratory tract cancers [7]. Transition metals in trace amounts are introduced during the manufacture and preparation of CNT. Given their oxidizable nature, studies suggest that metals including Fe, Co, and Ni are more toxic and fibrogenic upon their interaction with CNT as compared to pure CNT [89–93]. Vanadium pentoxide (V_2_O_5_), a transition metal byproduct of petrochemicals, is associated with fibrosis via generation of H_2_O_2_ and other ROS [94]. Occupational exposures to combustion-derived NP such as welding fumes consisting of metals such as Fe, Mn, Si, Cr, and Ni induce fibrogenic responses [95]. Metal containing welding fume NP elicited ROS-dependent lipid peroxidation and inflammation *in vivo* [96, 97].

## 5. Cellular Signaling Affected by Metal Nanoparticles

The prooxidant effects of NP result in the activation of signaling pathways, transcription factors, and cytokine cascade contributing to a diverse range of cellular responses. The regulation of redox homeostasis entails signaling cascades such as HIF-1, NF-*κ*B, PI3 K, and MAPK which control proliferation, metastasis, cell growth, apoptosis, survival, and inflammation [7, 12]. At an intermediate level of oxidative stress, proinflammatory pathways are activated in an attempt to maintain the redox equilibrium. The inflammatory cascade involves profibrotic mediators such as TNF-*α*, IL-1*β*, and TGF-*β* which have been implicated in the pathogenesis of fibrosis. Cells are known to counteract the overwhelming oxidative stress response via increased cytokine expression such as interleukins and TNF-**α**, activation of kinases, and inhibition of phosphatases thereby influencing the phosphorylation cascade. Protein phosphorylation is involved in the regulation of critical cellular responses including mitogenesis, cell adhesion, oncogenic transformation, and apoptosis. Thus, ROS response appears to be closely related to factors driving carcinogenesis [98]. 

### 5.1. NF-*κ*B

The NF-*κ*B group of proteins activates genes responsible for defense mechanisms against cellular stress and regulates miscellaneous functions such as inflammation, immune response, apoptosis, and cell proliferation. Prooxidant H_2_O_2_-mediated NF-*κ*B activation through the classical IKK-dependent pathway is well established. ROS such as OH^•^, HOCl, and ^1^O_2_ and RNS such as ONOO^−^ activate NF-*κ*B via the release of I*κ*Bs resulting in the nuclear translocation of NF-*κ*B [99, 100]. Once inside the nucleus, NF-*κ*B induces transcription of proinflammatory mediators resulting in inflammation and oxidative stress. During NP-mediated lung injury, ROS activate NF-*κ*B to modulate the production of proinflammatory TNF-*α*, IL-8, IL-2, and IL-6 from macrophages and lung epithelial cells [101]. Several metal oxide NP such as Zn, Cd, Si, and Fe exert their toxic effects via ROS-dependent NF-*κ*B activation [62, 102, 103].

### 5.2. AP-1

Activator protein (AP)-1 is a transcription factor activated in response to oxidants, cytokines, growth factors, and bacterial and viral infections. It is responsible for regulation of cell proliferation, differentiation, and apoptosis, thereby it is a key factor in carcinogenesis [104]. Activation of AP-1 under oxidative conditions is believed to be mediated via phosphorylation of protooncogene c-jun [68]. Metal NP including Cr, Ni, and Fe have been shown to activate AP-1 via ROS generation [60].

### 5.3. MAPK

MAPK are serine-threonine protein kinases that control a diverse range of cellular responses including proliferation, gene expression, differentiation, mitosis, cell survival, and apoptosis. MAPK consist of growth factor-regulated extracellular signal-related kinases (ERK) and the stress-activated MAPK, c-jun NH_2_-terminal kinases (JNK), and p38 MAPK. Once ROS production exceeds the capacity of the antioxidant proteins, free radicals may induce oxidative modification of MAPK signaling proteins (e.g., RTK and MAP3 K), thereby leading to MAPK activation. ROS may activate MAPK pathways via inhibition and/or degradation of MAPK phosphatases (MKP) [105, 106]. Finally, the site of ROS production and the concentration and kinetics of ROS production as well as cellular antioxidant pools and redox state are most likely to be important factors in determining the effects of ROS on activation of MAPK pathways [107]. Ag-NP activate JNK protein signaling and apoptosis in a variety of cells [50], whereas CeO_2_ NP trigger p38 MAPK signaling in bronchoalveolar cells [64].

### 5.4. PTP

Protein tyrosine phosphatases (PTP) are key regulatory components in signal transduction pathways involved in cell growth, differentiation, proliferation, and transformation. The highly reactive cysteine residues of PTP are predisposed to oxidative stress in the form of H_2_O_2_, free radicals or changes in intracellular thiol/disulfide redox state [98, 108]. Metal NP including Zn^2+^ and V^5+^ may be critical in redox regulation of PTP via the inhibition of MAPK and EGFR [109, 110].

### 5.5. Src

Src kinases belong to the nonreceptor tyrosine kinase family involved in the regulation of cell growth. Mild oxidative stress is sufficient to activate Src kinase which later triggers a cell signaling cascade [111]. This may explain the low dose of metal NP-induced lymphocyte cell death via ROS-dependent activation of Src kinases [112].

## 6. Carbon Nanotubes

One of the most promising materials in the field of nanotechnology is CNT, and their widespread applications are attributable to the diverse physical, chemical, and electrical characteristics they possess. CNT are high aspect ratio nanomaterials (HARN) having at least one of their dimensions in the order of 100 nm or less according to the British Standards Institute Report [113]. CNT are made of either single-walled (SW) or multiwalled (MW) graphite layers. With unique properties such as high tensile strength and conductivity, they have been explored in the areas of electronics, biotechnology, medicinal drug delivery, cosmetics, material science, and aerospace engineering. CNT structure facilitates their entry, deposition, and residence in the lungs and pleura, resulting in incomplete phagocytosis and clearance from the lungs [5]. Owing to their biopersistent and nonbiodegradable nature, and particularly their resemblance to needle-like asbestos fibers, CNT are believed to induce biologically harmful effects [89]. Physicochemical parameters such as particle size, surface modification, presence of metals, surface reactivity, and surface charge are responsible for the prooxidant effects of CNT. Frustrated phagocytosis of CNT has been implied in CNT-induced oxidative stress. 

## 7. Carbon Nanotube-Induced Oxidative Stress

One of the most frequently reported toxicity endpoints for CNT is the formation of ROS which can be either protective or harmful during biological interactions. Oxidative stress may be caused directly by CNT-induced ROS in the vicinity or inside the cell or could arise more indirectly due to the effects of internalized CNT on mitochondrial respiration [114] or in depletion of antioxidant species within the cell [64]. Moreover, NADPH-mediated ROS are critical for SWCNT-induced pulmonary responses [91]. The most likely mechanism for CNT-induced oxidative stress and lung toxicity involves mitochondrial dysfunction. Incomplete phagocytosis of CNT, presence of transition metals and specific reactive groups on the CNT surface are key drivers of ROS generation. Metal impurities such as Fe, Co, and Ni introduced within the CNT during their synthesis are key factors driving CNT-mediated ROS response [115, 116]. CNT-induced oxidative stress mediates important cellular processes including inflammation, cell injury, apoptosis, and activation of cellular signaling pathways such as MAPK and NF-*κ*B which are implicated in the pathogenesis of lung fibrosis [31, 117]. For instance, SWCNT dependent OH^•^ generation leads to activation of molecular pathways MAPK, AP-1, NF-*κ*B, and Akt associated with cell proliferation and tumor progression *in vitro* [93]. Several studies demonstrate SWCNT-induced oxidative stress [118–120]. Similarly, MWCNT exposures have been reported to induce ROS both *in vitro* and *in vivo* [18, 121–123]. Interestingly, oxidative stress is reported to be a mechanism for biodegradation of CNT. SWCNT undergoes oxidative biodegradation via myeloperoxidase, a prooxidant enzyme involved in host defense responses [120].  Table 2 summarizes the different studies that report ROS-dependent effects of CNT.

## 8. Role of ROS in CNT-Induced Inflammation

ROS and inflammation demonstrate an interdependent relationship in the case of exposure to NP. Inflammatory cells such as macrophages and neutrophils induce enormous ROS release in order to get rid of the NP. However, NP exposure-mediated oxidative stress leads to activation of RTK, MAPK, Akt, and NF-*κ*B contributing to the proinflammatory cascade [124]. Accordingly, CNT-induced ROS were reported to elicit pro-inflammatory transcription factors such as NF-*κ*B, AP-1 and MAPK *in vivo*. This was found to be an inflammation dependent response [93]. MWCNT treatment in macrophages mediates ROS-dependent activation of NF-*κ*B pathway, thereby inducing the expression of chemokines and cytokines such as TNF-*α*, IL-1*β*, IL-6, IL-10, and MCP-1 [18]. Likewise, MWCNT-induced nitrosative stress *in vivo* is associated with pulmonary inflammation [125].

## 9. Role of ROS in CNT-Induced Genotoxicity

CNT elicit genotoxic effects through direct interaction with DNA or indirectly via CNT-induced oxidative stress and inflammatory responses. CNT-induced sustained oxidative stress can result in DNA damage and abnormal cell growth, possibly leading to carcinogenesis and fibrogenesis [126, 127]. A plethora of studies demonstrate the genotoxic potential of both MWCNT and SWCNT [128–131]. ROS can activate cellular signaling pathways resulting in cell cycle arrest and apoptosis. CNT induce a multitude of genotoxic responses including DNA strand breakage, oxidation, micronuclei induction, chromosomal aberrations, formation of *γ*H2AX foci, and mutant frequencies [132]. Oxidative stress-dependent DNA breakage and repair and activation of signaling pathways including poly-ADP-ribose polymerase (PARP), AP-1, NF-*κ*B, p38, and Akt were reported in human mesothelial cells exposed to SWCNT [93]. CNT induce ROS-dependent lipid peroxidation both *in vitro* and *in vivo* [133, 134]. A number of studies account for mitochondrial membrane depolarization, damage, and oxidative stress upon CNT exposure [92, 135, 136]. Unlike the traditional prooxidant effect of NP, CNT have been reported to sequester ROS which in turn is associated with their structural defects [83]. This quenching is reported to be related to the genotoxic and inflammatory effects observed with CNT [137].

## 10. Role of ROS in CNT-Induced Fibrosis

Increased ROS has been implicated in lung inflammation and fibrosis. The inflammatory cascade is reported to contribute to oxidative stress mediated lung injury [138]. Exposure to CNT results in expression of genes responsible for inflammation and fibrosis via the activation of cell signaling pathways and transcription factors including NF-*κ*B, STAT-1, MAPK, and RTK [31]. ROS-dependent p38-MAPK has been shown to be responsible for CNT-induced collagen and angiogenic responses [118]. Additionally, SWCNT induce fibrogenic effects via ROS-mediated NF-*κ*B activation [139], whereas MWCNT induce fibroblast to myofibroblast differentiation via ROS-dependent NF-*κ*B activation [18]. 

## 11. Oxidative Stress as an Underlying Mechanism for NP Toxicity

**Figure 1:**
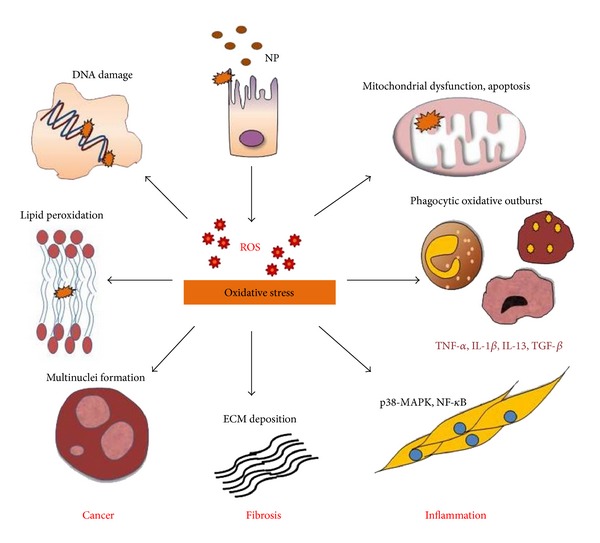
Prooxidant pathway for NP-induced toxicity: various NP exhibit oxidative stress dependent toxicity. Upon NP exposure, ROS generation is capable of inducing oxidative DNA damage, strand breaks, protein denaturation, and lipid peroxidation thereby demonstrating the mutagenic and carcinogenic characteristics associated with NP. Excess free radical production leads to mitochondrial membrane damage causing necrosis and cell death. Phagocytes including neutrophils and macrophages generate massive ROS upon incomplete phagocytosis of NP through the NADPH-oxidase enzyme system whereas NP-induced ROS triggers an inflammatory cascade of chemokine and cytokine expression via activation of cell signaling pathways such as MAPK, NF-*κ*B, Akt, and RTK. Furthermore, oxidative stress mediated stimulation of these cellular mechanisms results in transcription of genes responsible for fibrosis, EMT, and carcinogenesis. NP-elicited ROS is at the center stage for majority of the ensuing adverse outcomes.

Findings from several studies have pointed out that ROS generation and oxidative stress occur as an early event leading to NP-induced injury. Oxidative stress corresponds with the physicochemical reactivity of NP including metal-based particles as well as the fibrous CNT. Oxidative stress related to NP exposure involves mitochondrial respiration, mitochondrial apoptosis, activation of NADPH oxidase system, alteration of calcium homeostasis, and depletion of antioxidant enzymes; all of which are associated with tissue injury. NP-driven ROS response contributes to activation of cell signaling pathways, inflammatory cytokine and chemokine expressions, and specific transcription factor activation. Activation of these cellular mechanisms is closely associated with transcription of genes involved in inflammation, genotoxicity, fibrosis, and cancer. Thus, the pathological consequences observed during NP exposure could be attributable to ROS generation. It is essential to incorporate these adverse biological responses as a screening tool for toxic effects of NP. For instance, over-expression of antioxidant enzymes is indicative of the mild oxidative stress, whereas mitochondrial apoptosis occurs during conditions of toxic oxidative stress. The hierarchical model of ROS response provides a scale to gauge the adverse health effects upon NP exposures. A NP exposure study must collectively involve rigorous characterization of NP and assign *in vitro* and *in vivo* oxidative stress markers as toxicity endpoints as a predictive paradigm for risk assessment [6, 9, 12].  Figure 1 summarizes the key findings regarding the oxidative effects of NP and resulting toxicity.

## 12. Conclusion

This paper reviews the cellular mechanisms of NP-induced oxidative stress and toxicity. We focus on the toxicity of metal oxide NP and CNT with respect to the oxidative stress paradigm. The principal factors for NP-induced oxidative stress involve (a) the oxidative properties of the NP themselves and (b) oxidant generation upon interaction of NP with cellular material. The direct prooxidant effects of NP are attributable to their physicochemical properties including surface reactivity, particle size, surface charge, chemical composition, and the presence of transition metals. Therefore, it is necessary to ensure extensive characterization of the physicochemical properties for safer design and manufacture of NP. Whereas, ROS mediated via NP-cell interaction involve mechanisms including immune cell activation, mitochondrial respiration, and NADPH oxidase system. Apart from ROS, NP also arbitrate RNS-mediated injury. Given their chemical reactivity, metal-based NP induce oxidative damage to cellular macromolecules such as proteins, lipids, and DNA via Fenton-type and Haber Weiss-type reactions. The key pathophysiological outcomes of oxidative insults during metal NP exposures involve cell membrane damage, lipid peroxidation, protein denaturation, and alteration of calcium homeostasis. Furthermore, the findings in the review article suggest that CNT-induced oxidative stress is indicative of the pulmonary toxicity of CNT. Metal-based NP and fibrous CNT-mediated ROS result in activation of cell signaling pathways, transcription factor activation, cytokine mediator release, and apoptosis. The persistent activation of these signaling cascades has some clinical ramifications. Redox imbalance via engineered NP exerts undesirable pathophysiological outcomes such as genotoxicity, inflammation, fibrosis, and carcinogenesis. It is of utmost importance to understand the molecular and cellular mechanisms of NP-induced oxidative stress which in turn will yield novel strategies to mitigate the toxicity of engineered NP. Moreover, it necessitates the establishment of stringent procedures for testing the oxidative potential of manufactured NP prior to their commercialization. Identifying the major cellular targets for NP-induced ROS will facilitate safer design and manufacture of NM in the market place.

## Figures and Tables

**Table 1:** List of studies describing the ROS-dependent effects of metal-based NP.

Nanoparticles	ROS-dependent effect	Reference
Iron oxide		
Iron oxide	Necrosis and apoptosis in murine macrophage (J774) cells	[61]
Zero-valent iron	Acute cytotoxicity in human bronchial epithelial cells	[140]
Iron oxide	Human microvascular endothelial cell permeability	[141]
SPIONS	Activation of NF-*κ*B and AP-1, inflammation in human epidermal keratinocytes (HEK) and murine epidermal cells (JB6 P(+))	[142]

Copper oxide		
Copper oxide	Genotoxicity in human lung epithelial cells	[143]
Copper oxide	Mitochondrial dysfunction, oxidative DNA damage, cell death in A549 cell line	[144]
Copper oxide	Cytotoxicity *in vitro* in Hep-2 cell line	[145]
Copper oxide	Nephrotoxicity and hepatotoxicity *in vivo *	[146]

Cerium oxide		
Cerium oxide	Lung inflammation and alveolar macrophage apoptosis *in vivo *	[147]
Cerium oxide	Apoptosis via caspase-3 activation and chromatin condensation *in vitro* in BEAS-2B cells	[64]
Cerium oxide	HO-1 induction via the p38-Nrf-2 signaling pathway *in vitro* in BEAS-2B cell line	[148]
Cerium oxide	Lipid peroxidation and membrane damage *in vitro* in lung cancer cells	[149]

Zinc oxide		
Zinc oxide	Mitochondrial dysfunction, morphological modification, and apoptosis *in vitro* in human fetal lung fibroblasts	[59]
Zinc oxide	Cellular oxidant injury, excitation of inflammation, and cell death in BEAS-2B and RAW 264.7 cells	[150]
Zinc oxide	Mitochondrial damage, apoptosis, and IL-8 release *in vitro* in LoVo human colon carcinoma cell line	[151]
Zinc oxide	Mitochondrial damage, genotoxic and apoptotic cell effects *in vitro* human liver cells	[152]
Zinc oxide	Genotoxic and apoptotic responses *in vitro* in human skin melanoma cell line (A375)	[153]
Zinc oxide	Endoplasmic reticulum stress, apoptosis, and necrosis in rat retinal ganglion cells	[154]
Zinc oxide nanorods	Apoptosis in human alveolar adenocarcinoma cells via p53, surviving, and bax/bcl-2 pathways	[155]

Nanosilica		
Nanosilica	Cytotoxicity and apoptosis via activation of p53 and Bax *in vitro* in human hepatic cell line p53 and p21 mediated G1 phase arrest *in vitro* myocardial H9c2 (2-1) cells	[156] [157]
Nanosilica	Cell cycle arrest and apoptosis *in vitro* human embryonic kidney cell line	[158]
Nanosilica	Hepatotoxicity *in vitro* in Kupffer cells and ROS-mediated cell death, oxidative DNA damage	[159]

Nickel oxide		
Nickel oxide	Lipid peroxidation, apoptosis *in vivo* in human epithelial airway cells	[160]
Nickel ferrite	Apoptosis in A549 cells through oxidative stress via p53, survivin, bax/bcl-2, and caspase pathways in normal Chang (normal human liver), MCF10A (normal breast epithelial), and WI38 (normal lung fibroblast) cell lines	[161]

Titanium dioxide		
Titanium dioxide	Apoptotic cell death through ROS-mediated Fas upregulation and Bax activation	[162]
Titanium dioxide	Cytotoxic and genotoxic effects *in vitro* in human amnion epithelial (WISH) cell line	[163]
Titanium dioxide	Cytotoxicity and apoptotic cell death *in vitro* in HeLa cell line	[164]

Aluminum oxide		
Aluminium oxide	Mitochondria mediated oxidative stress and cytotoxicity in human mesenchymal stem cells	[165]

Gold		
Gold	Lipid peroxidation and autophagy *in vitro* in MRC-5 lung fibroblasts	[166]

Silver		
Ag-NP	Mitochondrial damage and genotoxicity in human lung fibroblast cells (IMR-90) and human glioblastoma cells (U251)	[167]
Ag-NP	JNK-mediated mitochondrial apoptosis in NIH3T3 fibroblasts	[50]
Ag-NP	Mitochondrial damage, apoptosis *in vitro* in A549 cells	[168]

Cobalt-chromium (Co-Cr)		
Co-Cr NP	Oxidative DNA damage, micronuclei induction, reduced cell viability in human dermal fibroblasts	[169]

**Table 2:** List of studies describing the ROS-dependent effects of CNT.

	CNT	
SWCNT with 30% iron by mass	Lipid peroxidation, reduced cell viability, and antioxidant reserve in human keratinocytes	[170]
Acid treated MWCNTs with Co and Ni	Decreased cell viability, altered mitochondrial membrane potential in rat macrophages (NR8383) and human A549 lung cells	[92]
SWCNT	Reduced cell viability and antioxidant reserve in rat lung epithelial cells	[171]
SWCNT	Increased apoptosis, DNA damage, activated MAPKs, AP-1, NF-*κ*B, and Akt in normal and malignant human mesothelial cells	[93]
SWCNT	Reduced cell proliferation, activation of NF-*κ*B in human keratinocytes	[119]
Unpurified SWCNT (30% w/w iron)	Activation of AP-1 and NF-*κ*B, cytotoxicity, and proinflammatory response *in vitro* and *in vivo *	[172]
Unpurified SWCNT (17.7% w/w iron)	Lipid peroxidation, acute inflammatory response, decreased respiratory function in adult C57BL/6 mice	[91]
Raw MWCNT	Dose-dependent cytotoxicity in RAW 264.7 macrophages and A549 cells: cell inflammation, membrane leakage, lipid peroxidation, and protein release	[173]
MWCNT	Increase in cell permeability, cell migration, and endothelial permeability in human microvascular endothelial cells (HMVEC)	[174]
SWCNT	Activation of p38 MAPK in CNT mediated fibrogenic and angiogenic responses *in vitro* in human lung fibroblasts	[118]
MWCNT	Activation of NF-*κ*B, fibroblast-myofibroblast transformation, profibrogenic cytokine, and growth factor induction *in vitro* (BEAS-2B, WI-38, and A549 cell lines)	[18]

## Abbreviations

ROS: Reactive oxygen species
NP: Nanoparticles
NM: Nanomaterials
RNS: Reactive nitrogen species
CNT: Carbon nanotubes
H_2_O_2_: Hydrogen peroxide
O_2_^•−^: Superoxide anion
OH^•^: Hydroxyl radical
^1^O_2_: Singlet oxygen
HOCl: Hypochlorous acid
ONOO^−^: Peroxynitrite
AM: Alveolar macrophages
NADPH: Nicotinamide adenine dinucleotide phosphate
Nrf2: Nuclear factor (erythroid-derived 2)-like 2
MAPK: Mitogen activated protein kinase
NF-*κ*B: Nuclear factor kappa-light-chain enhancer of activated B cells
iNOS: Inducible nitric oxide synthase
IL-1*β*: Interleukin-1beta
ERKs: Extracellular signal-related kinases
GSH: Glutathione
GSSG: Glutathione disulfide
8-OHdG: 8-Hydroxyl-2′-deoxyguanosine
AP-1: Activator protein-1
STAT-1: Signal transducer and activator of transcription-1
RTK: Receptor tyrosine kinases
PTP: Protein tyrosine phosphatases
HARN: High aspect ratio nanomaterials
PARP: Poly-ADP-ribose polymerase
TNF-*α*: Tumor necrosis factor-alpha
TGF-*β*: Transforming growth factor-beta
EMT: Epithelial-mesenchymal transition.
